# Single-mode, high-power, mid-infrared, quantum cascade laser phased arrays

**DOI:** 10.1038/s41598-018-33024-7

**Published:** 2018-10-05

**Authors:** Wenjia Zhou, Donghai Wu, Quan-Yong Lu, Steven Slivken, Manijeh Razeghi

**Affiliations:** Center for Quantum Devices, Department of Electrical Engineering and Computer Science, Northwestern University, Evanston, IL 60208 USA

## Abstract

We demonstrate single-mode, 16-channel, optical phased arrays based on quantum cascade laser technology, with emission wavelengths around 4.8 µm. The integrated device consists of a distributed feedback seed section, a highly-efficient tree array multi-mode interferometer power splitter, and a 16-channel amplifier array with a 4° angled facet termination. With a single layer Y_2_O_3_ coating, the angled facet reflectivity is estimated to be less than 0.1% for suppressing amplifier self-lasing. A peak output power of 30 W is achieved with an emission spectrum narrower than 11 nm and a side mode suppression ratio over 25 dB. Far field distribution measurement result indicates a uniform phase distribution across the array output. Using the same phased array architecture, we also demonstrate single-mode 3.8 µm QCL amplifier arrays with up to 20 W output power.

## Introduction

Optical phased arrays (OPAs) are emerging as important light sources for non-mechanical beam steering. In the past few years, extensive research has been done on near infrared OPAs, which was heavily motivated by light detection and ranging (LIDAR) applications^1–3^. However, there have been limited demonstrations of OPAs in the mid-infrared (3–12 µm) spectral region, which is critical for chemical and biological sensing, free-space communications and infrared-countermeasure applications^4^. For all of these applications, a beam steerable and high-power laser source is highly desirable.

Quantum cascade lasers (QCLs) have become the leading laser sources in the mid-infrared, thanks to their compact form, ability to operate at room temperature, and high wavelength tunability^5–7^. Since QCLs are based on semiconductor laser technology, they are extremely suitable for photonic integrated circuits development. Using on-chip photonic integration, we had previously demonstrated monolithically widely tunable QCL sources by combining the outputs of an eight-element sampled grating distributed feedback (SGDFB) laser array into a single emitting aperture^8,9^. For the reverse process of beam combining, splitting the output of a single laser into a laser array would create an optical phased array, which could be utilized for increased power output and non-mechanical beam steering. In the case of non-mechanical beam steering or spectroscopy applications, it is also advantageous to have a narrow bandwidth or single-mode seed laser. For splitting the output of the seed laser into a large format array, a low loss beam splitter design is necessary. In ref.^9^, a tree-array Y-junction and funnel combiner is used for maximizing power throughput and to control the transverse mode quality. However, the power throughput is only 7% on average for three stages of beam combining due to the large bandwidth of the widely tunable device. In the case of a single-mode device, an excellent choice for beam splitting is the multimode interferometers (MMI), which has long been used for near-infrared beam splitting and only recently demonstrated in the mid-infrared region with high splitting efficiency up to 94%^10^. In this work, we utilize on-chip integration of a DFB seed laser, a tree-array MMI power splitter, and an optical amplifier array in order to demonstrate single-mode, 16-channel, λ = 4.8 µm mid-infrared OPAs. Besides demonstration of a stable phase relationship between all emitters, this technology also delivers very high output power up to 30 W thanks to the large available active area of the amplifier array. In addition, compared to single mode photonic crystal DFB QCLs^11^ and master-oscillator power-amplifier (MOPA) type QCLs^12^, the OPA design has the advantage of operating at higher duty cycle due to the separation of amplifier elements. Furthermore, the demonstrated OPA structure is translational in wavelength; using the same design architecture and fabrication process, a single-mode λ = 3.8 µm phased array, which is suitable for spectroscopy sensing of greenhouse gas N_2_O, is demonstrated with up to 20 W of output power.

## Results

### Single-mode optical phased array design

**Figure 1:**
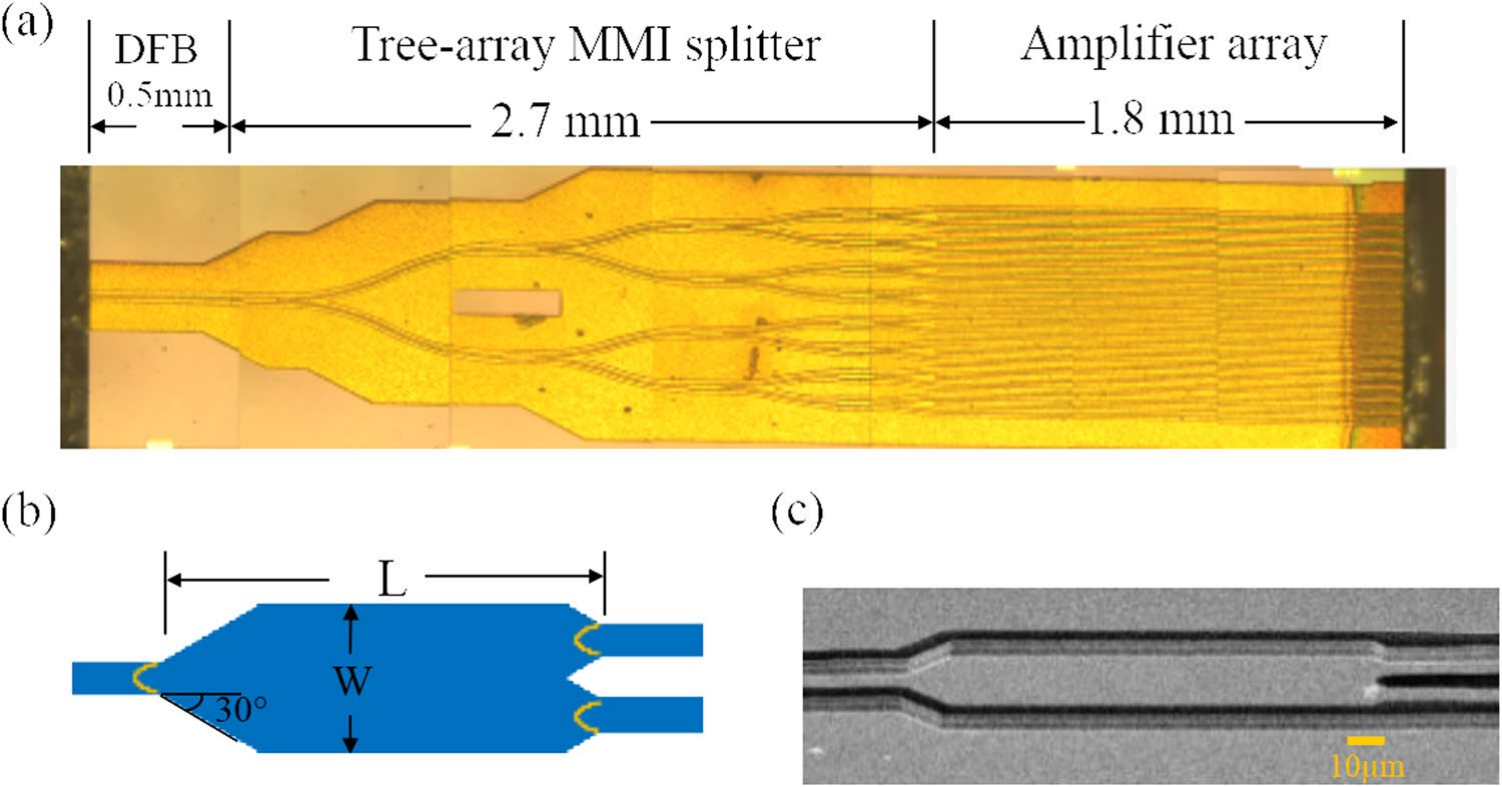
Single mode 16-channel optical phased array. (**a**) Composite microscope image of the phase-locked quantum cascade amplifier (QCA) array integrated with a distributed feedback (DFB) seed section, and a tree array multimode interferometer (MMI) based power splitter. (**b**) Schematic structure of a 1 × 2 MMI splitter (not to scale). (**c**) Scanning electron microscope (SEM) image of a fabricated MMI splitter.

Figure 1(a) shows the composite optical microscope image of the OPA source designed for 4.8 µm emission. The device is monolithic and has a compact size of 5 mm × 1 mm. The DFB seed section is 0.5 mm long and is used to provide a single mode input into the amplifier array. First order DFB grating with a duty cycle of nearly 50% and grating period of 755 nm is patterned onto the grating layer. The simulated refractive index for the etched and un-etched part of the grating is 3.17 and 3.194 respectively, and the coupling strength is therefore

$$\kappa =\frac{1}{{\rm{\Lambda }}}\cdot \frac{{\rm{\Delta }}n}{{n}_{eff}}=100\,{{\rm{c}}{\rm{m}}}^{-1},$$

where Λ is the grating period, *Δn* and *n*_*eff*_ are the refractive index step and the effective refractive index. The product of the DFB section length and coupling strength *κ*L is 5, which is a moderate number for achieving a good output power and mode discrimination^13^. The output of the DFB seed is evenly split into a 16-channel amplifier array, which has a ridge width of 6 µm and a separation (pitch width) of 50 µm. A tree array of 1 × 2 MMIs connected by S-bend waveguides, which have a 1000 µm bending radius for achieving a low loss and minimal beam degradation in the curved waveguides, is used for power splitting. Respectively, Fig. 1(b,c) shows the schematic structure and scanning microscope (SEM) image of a W=20 µm wide 1 × 2 MMI for power splitting. The optimal MMI length for in-phase supermode beam splitting is

$${\rm{L}}={{\rm{n}}}_{{\rm{eff}}}{{{\rm{W}}}_{{\rm{eff}}}}^{{\rm{2}}}/2{\rm{\lambda }},$$

where W_eff_ is effective width of the MMI^14^. The effective MMI width, which takes into account the transverse mode penetration into surrounding SiO_2_ layer, is W_eff_ = W + λ/π/(n_eff_^2^ − n_SiO2_^2^)^1/2^ = 20.5 µm for a standard, TM-polarized QCL ridge waveguide. Based on W_eff_, the calculated optimum MMI length is 139 µm for n_eff_ = 3.18 and λ = 4.8 µm. As has been shown in ref.^10^, at the optimum MMI length for the in-phase supermode beam splitting, power transmission of the out-of-phase supermode is close to zero, which indicates a nearly 100% reflection. For suppressing total reflection of the out-of-phase supermode, the input and output sides of the MMI are tapered by 30°, as shown in Fig. 1(b). The 30° angle is chosen to be larger than the beam divergence angle of the 4.8 µm laser emitted from the d = 6 µm wide waveguide which equals 2λ/n_eff_πd = 11°.

**Figure 2:**
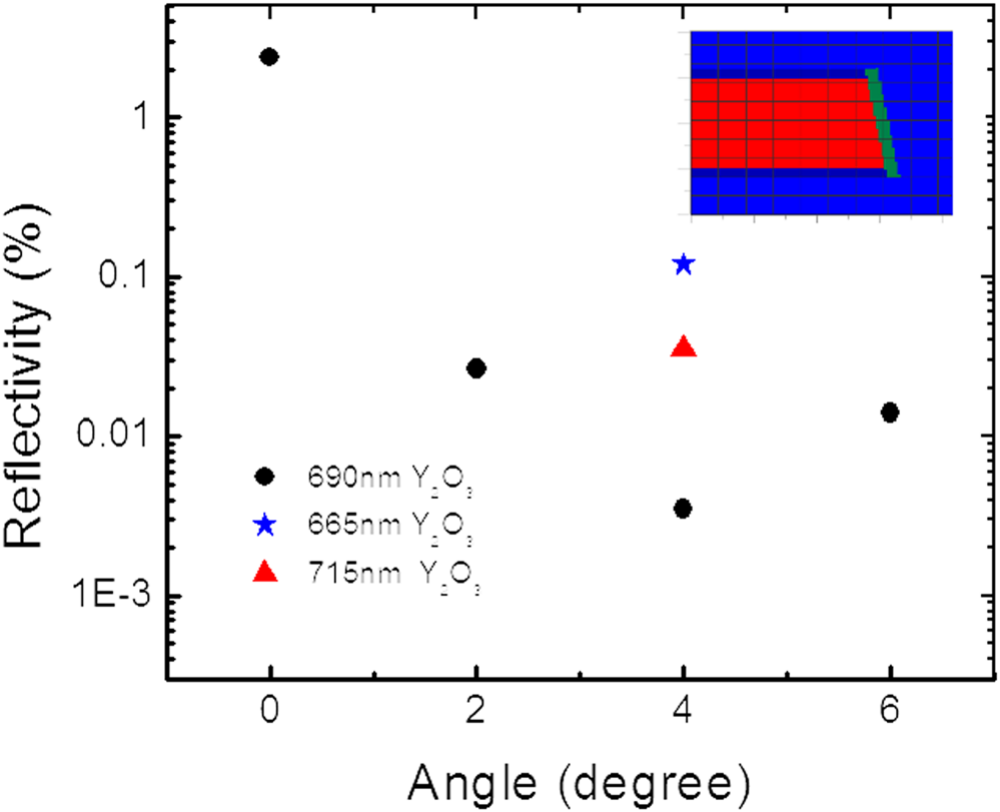
Angled facet termination design. Simulated facet reflectivity as a function of termination angle with different coating conditions; the inset shows simulated refractive index distribution of a horizontal slice through the AR-coated, angled waveguide.

In order to have single mode emission, self-lasing of the amplifier array needs be suppressed, which requires an ultra-low reflectivity on the laser front facet. Anti-reflection(AR) coating of QCLs had usually been done by depositing a quarter-wavelength dielectric film, such as Y_2_O_3_, on the facet. Normally, a single layer Y_2_O_3_ could provide a reflectivity below 1% in a narrow band. To reach an even lower reflectivity and to enhance the reflectivity tolerance to coating thickness variation, we have designed an angled facet termination for the amplifier array, which had been demonstrated on near-infrared semiconductor laser amplifiers^15^. For the angled facet design, the facet reflectivity is significantly reduced due to decreased overlap between the reflected laser beam and the input beam. When AR-coated, there are certain angles that have a local reflectivity minimum because of the destructive interference between beams reflected to the laser cavity form the two sides of the coating material. The reflectivity of the AR-coated angled facet is simulated by a rigorous 3D finite-difference time-domain (FDTD) method. Figure 2 shows the facet reflectivity as a function of the termination angle under various coating conditions. It suggests that a reflectivity below 0.01% is achievable for a 4° angle with 690 nm Y_2_O_3_ coating. Even with a coating thickness variation of ±25 nm, which could be well controlled by ion beam deposition, the reflectivity is still below 0.1%. As a result, the 4° angle termination was chosen for the amplifier array.

### 4.8 µm single-mode OPA result

**Figure 3:**
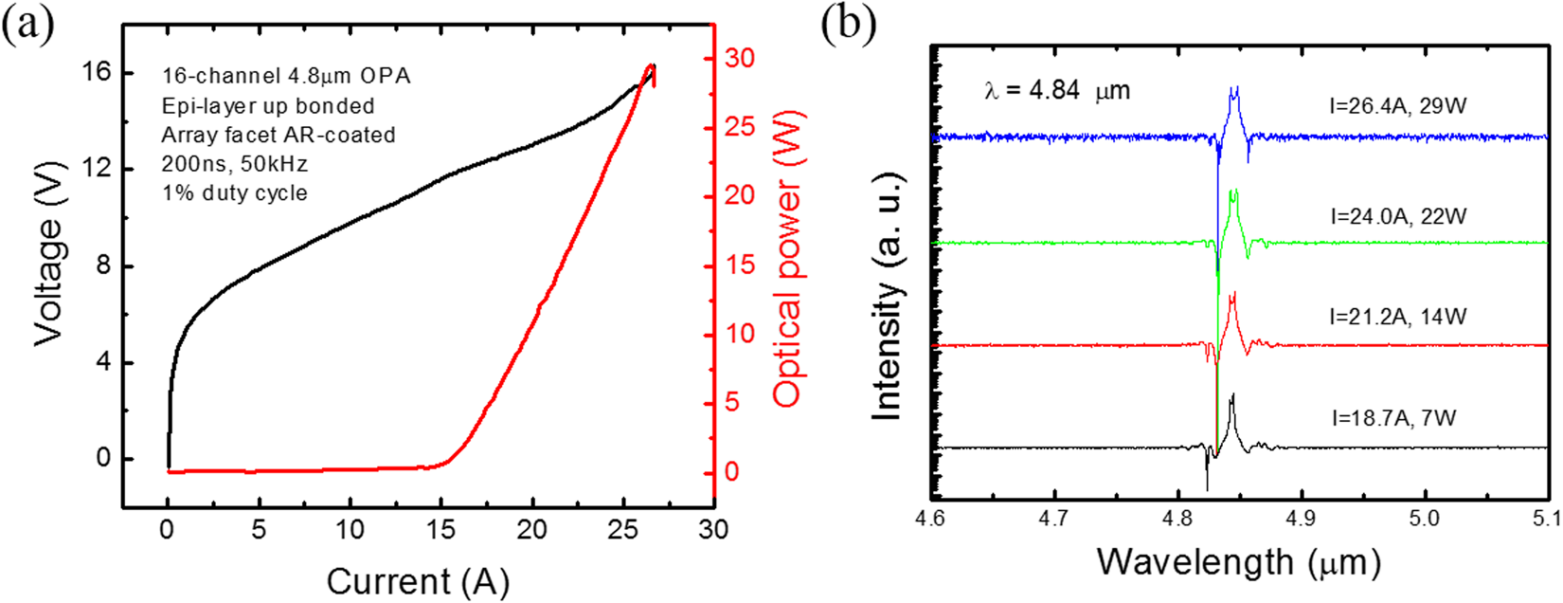
P-I-V and spectrum measurement result of 4.8 µm 16-element OPA. (**a**) Power-current-voltage (PIV) curves of a 16-channel 4.8 µm single mode phase-locked QCA array under pulsed mode operation (1% duty cycle). (**b**) Spectrum measurement result of the QCA array under different current conditions plotted in logarithmic scale. Spectra are offset vertically for clarity.

The λ~ 4.8 µm QCL core structure is similar to the wafer used in ref.^16^, which is of a temperature insensitive design with inserts of AlAs and close-to-lattice-matched Ga_0.47_In_0.53_As and Al_0.48_In_0.52_As. After materials growth and device fabrication (see Methods), individual laser dies of 5 mm length, with front facet AR-coated with 690 nm Y_2_O_3_, were epi-layer up bonded to copper submounts using indium solder and tested at a heat sink temperature of 15 °C (see Methods). Figure 3(a) shows the power-current-voltage (P-I-V) characteristics of a 16-channel phase-locked quantum cascade amplifier (QCA) array under pulsed mode operation. For simplicity, the device is homogeneously pumped and there is no electric isolation between the DFB seed and the amplifier array. The reported output power was measured exclusively from the multi-aperture (array) output facet. With epi-layer up bonding, 1% duty cycle operation, and a pulse width of 200 ns, a peak power of 30 W was obtained. The lasing current threshold is 15 A (4.9 kA/cm^2^), and the slope efficiency is 2.72 W/A. In comparison to a 5 mm long, 6 µm wide Fabry-Perot (FP) laser, which is fabricated by the same process and emits up to 4.7 W (two facets), the output power enhancement factor is 6.4, while the area ratio of the OPA to the FP laser is 9.4. The FP laser has a current threshold density of 2.9 kA/cm^2^, which is lower than the OPA device and is partially because the FP laser is uncoated. It is also possible that the DFB seed laser, which has an ultra-lower reflectivity front facet, is under coupled. This means there is potential for increasing the phased array output power by optimizing the length and coupling coefficient of the DFB section. Also, since there is no gain saturation observed up to the rollover input current, higher output power could also be obtained by increasing the length of the amplifier array.

Figure 3(b) shows the emission spectrum of the OPA measured at different current conditions (see Methods). Near the lasing threshold (I = 1.25I_th_), the full width at half maximum(FWHM) of the spectrum is only 4 nm. Near the rollover input current of I = 26.4 A, the FWHM of the spectrum is only 7 nm. There is a slightly dual-peak behavior with a spacing of 2.5 cm^−1^ observed. Since the spacing is much narrower than the *κ*/n_eff_π = 10 cm^−1^ photonic bandgap of the DFB grating, the possibility of simultaneous lasing on both sides of the bandgap is excluded. We believe the slightly dual-peak behavior is caused by intra-pulse heating, as has been observed in ref.^17^, and can be removed once continuous wave (CW) operation of the OPA is realized. In all current conditions, the side-mode suppression ratio (SMSR) is more than 25 dB. The high quality single mode emission behavior indicates a low reflectance front facet which is a result of the AR-coated angled facet design.

**Figure 4:**
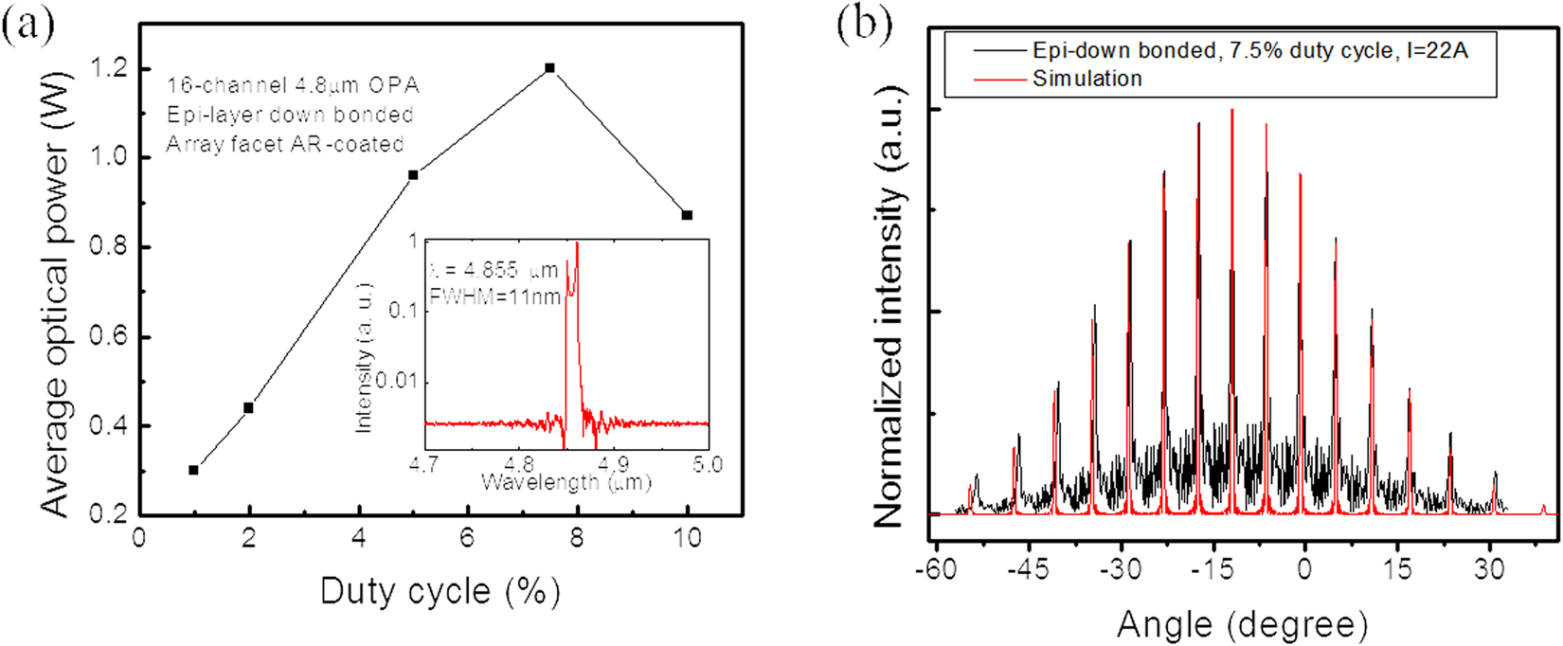
Measurement result of an epi-layer down bonded OPA. (**a**) Average power of an epi-layer down bonded 16-channel OPA as a function of pulsed operation duty cycle. The inset shows the emission spectrum of the device operating at the roll-over current under 7.5% duty cycle. (**b**) Far field distributions of the 16-channel, single mode phase-locked array operating at the rollover current under 7.5% duty cycle.

The device architecture is also suitable for high duty cycle operation thanks to the narrow ridge width of the amplifier array and their 50 µm separation. One laser die was epi-layer down bonded to a diamond submount prior to being bonded to a copper submount for testing at high duty cycles. Figure 4(a) shows the maximum average output power achieved as a function of the duty cycle. At 7.5% duty cycle, an average output power of 1.2 W is achieved. Above the 7.5% duty cycle, average power starts to drop due to thermal loading effects. Future design improvements, such as a wider array pitch or lower laser core doping, could result in significantly higher average power. Nevertheless, being able to deliver watt level average power makes the laser source highly suitable for stand-off spectroscopy sensing applications. The inset of Fig. 4(a) shows the emission spectrum of the device operating at the roll-over current under 7.5% duty cycle. The peak width is only 11 nm, and the SMSR is over 25 dB.

Far-field distribution of the epi-layer down bonded OPA was measured using a liquid nitrogen-cooled mercury-cadmium-telluride (MCT) detector with an aperture-limited angular resolution of 0.1°. The laser was mounted on a computer-controlled rotation stage at a distance of ~70 cm away from the detector to avoid detector saturation and also ensure the high angular resolution condition. Fig. 4(b) shows the measured far field distribution of the device operating at the rollover current under 7.5% duty cycle. It can be clearly seen that the far field distribution has a Gaussian shape envelope and has modulation peaks which indicates phase-locking behavior of the array output. The center of the far field radiation envelope is at *θ*_*c*_ = −12°, which is in agreement with the 4° angled facet design since $|sin{\theta }_{c}|={n}_{eff}\cdot sin(4^\circ )$. The far field radiation pattern can be interpreted by the 16-slit Fraunhofer diffraction theory. As shown in Fig. 4(b), an excellent match is achieved between the measurement result and simulation. Specifically, the maxima of side lobes occurs when sin(*θ*_m_ − *θ*_c_) = mλ/d, m = 0, ±1, ±2, …, where d is the pitch size. As a result, the interference peak interval is sin^−1^(λ/d) = 5.55°, when *θ*_m_ − *θ*_c_ is small. From the measurement result, the interval of the interference peaks is about 5.5°. Interference peak width is calculated as 2sin^−1^(λ/16 d), and equals 0.69°. Around −12°, the full width of the interference peak is 0.8°, which is close to the theoretical prediction.

### 3.8 µm single-mode OPA result

**Figure 5:**
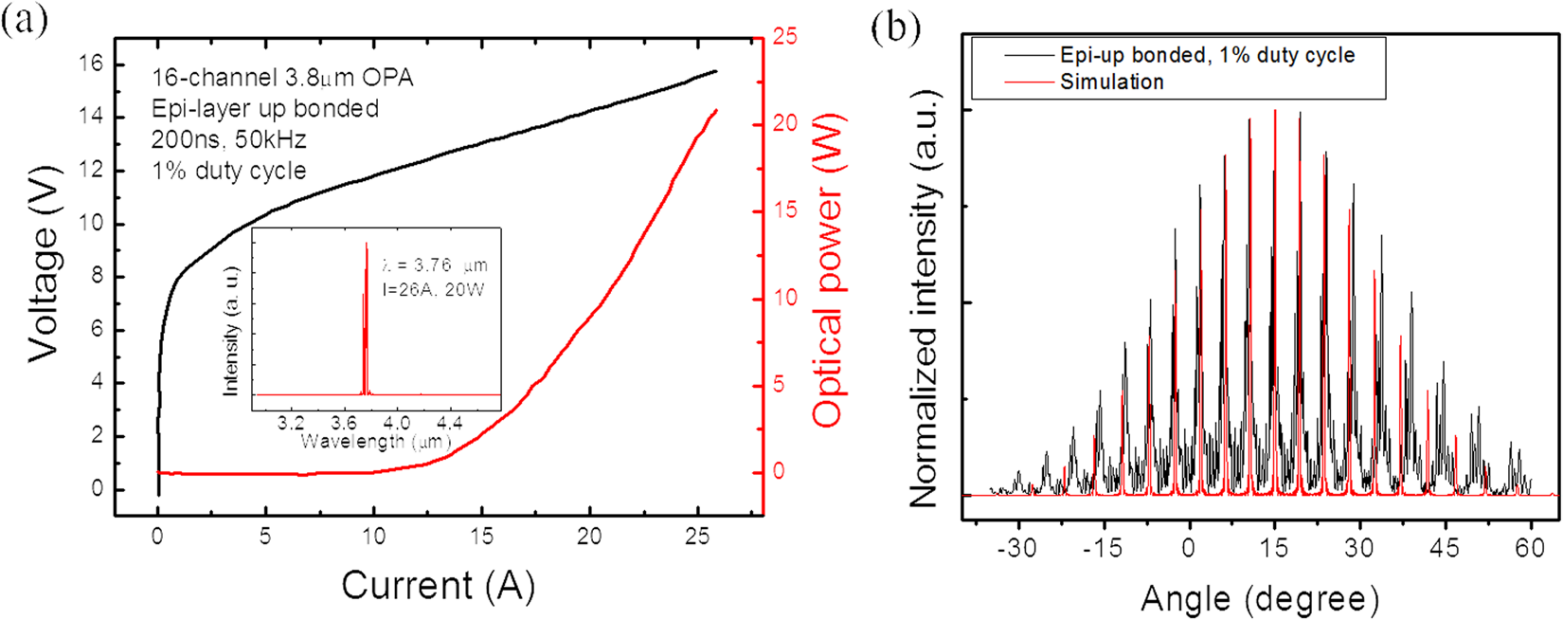
P-I-V, spectrum and far field measurement result of 16-element 3.8 µm OPA. (**a**) Power-current-voltage (PIV) curves of a 16-channel 3.8 µm single mode phase-locked QCA array under pulsed mode operation (1% duty cycle). The inset shows the emission spectrum of the device operating at the roll-over current (**b**) Far field distributions of the OPA operating near the maximum input current.

Since the demonstrated single-mode phased array is adaptive in wavelength, we apply the same architecture for realizing high-power 3.8 µm single mode OPAs. The 3.8 µm device corresponds to an absorption line in the infrared spectrum of N_2_O, which is an important trace gas that potentially contributes to global warming. The QCL core structure used for this demonstration is based on highly strain-balanced Ga_0.21_In_0.79_As/Al_0.77_In_0.23_As superlattice^18^. 30 stages of laser core with a 300 nm thick grating layer were grown by gas-source molecular beam epitaxy (GSMBE). The buried grating structure provides an estimated coupling strength of 133 cm^−1^. A DFB section length of 1 mm is utilized to obtain a lower current threshold. The laser ridge width is chosen as 5 µm to support fundamental mode operation and the amplifier array also has a pitch width of 50 µm. Each 1 × 2 MMI splitter is 16 µm wide and 120 µm long. Fabrication of the 3.8 µm OPAs is similar to that of the longer wavelength OPA (see Methods). As shown in Fig. 5(a), a maximum output power of 20 W was achieved, and single mode operation with a SMSR over 25 dB was obtained at the maximum input current. Figure 5(b) shows the far field distribution measurement result, which matches well with the simulated far field distribution of a laser array with a uniform phase profile across the array output. The interference peak interval is 4.5°, which is smaller compared to the 4.8 µm OPA because of the shorter wavelength.

## Discussion

In conclusion, we have demonstrated single mode, 4.8 µm, 16-channel, optical phased arrays. Up to 30 W single mode peak output power was obtained. Under epi-layer down bonding configuration and 7.5% duty cycle operation, a maximum average power of 1.2 W was achieved with single mode emission narrower than 11 nm and SMSR of 25 dB. Also, in-phase supermode operation has been demonstrated up to the rollover current. Using the same device architecture, we have also demonstrated 3.8 µm single-mode, 16-channel, OPAs with up to 20 W output power. Compared to single mode high-power photonic crystal DFB QCLs and MOPA type QCLs, the OPA devices deliver a much higher average power and has huge potential for high power CW operation under proper thermal management. In the future, the DFB laser could be replaced by a SGDFB laser to achieve a wide tunability^19^. Furthermore, monolithic beam steering could be achieved through active (electrical) phase control of the individual array outputs. Also, the output of the arrays could be coupled to silicon photonic chips for passive beam steering or combining^20,21^, which will open new possibilities for mid-infrared photonics.

## Methods

### Growth and fabrication

The MBE growth of the λ~ 4.8 µm wafer started with a 1 μm n-doped (N_d_=2 × 10^16^ cm^−3^) InP buffer layer and a 30-stage QCL core designed for emission at λ = 4.8 μm. A 100 nm InP spacer layer and a 300 nm thick GaInAs grating host layer were grown directly on top of the emitting stages. DFB gratings with a period of 755 nm were patterned into the grating host layer using e-beam lithography and plasma etching. After grating patterning, a metal-organic chemical vapor deposition (MOCVD) regrowth of 3 µm low-doped InP cladding (Si, ~2 × 10^16^ cm^−3^) and 1 µm high-doped InP cap layer (Si, ~5 × 10^18^ cm^−3^) was performed. After regrowth, a 1000 nm thick SiO_2_ mask was deposited with plasma enhanced chemical vapor deposition (PECVD). A double channel waveguide pattern was first defined by a photolithography process and then transferred to the SiO_2_ layer. The laser waveguide was subsequently etched to 8 µm depth using an inductively coupled plasma (ICP) dry etching technique with a chemistry of ‘Cl_2_/Ar/H_2_’, which ensures a good control over MMI dimensions and the curved waveguide. After the removal of the SiO_2_ mask in buffered hydrofluoric acid, another 1000 nm of SiO_2_ was deposited by PECVD for waveguide passivation. After window opening on top of the laser ridge, a top contact was formed after Ti/Au deposition. A 3 µm gold layer was electroplated on the top surface. After polishing the substrate to 150 µm, a AuGe/Ni/Au bottom contact was evaporated. The growth and fabrication of the 3.8 µm device is similar to the 4.8 µm one, except that the laser core is designed for λ = 3.8 µm emission, and the DFB grating period is of 595 nm.

### Device Testing

Laser dies were either bonded epi-layer up to copper submounts with indium solder directly or bonded epi-layer down to a diamond heat spreader first before bonding to copper submounts. Laser submounts were mounted on a temperature controlled-stage and held at a temperature of 288 K. All devices were tested under pulsed mode. For output power measurement, a calibrated thermopile detector was used to measure the average power, and the peak power was obtained from the measured average power and the known duty cycle. Device output wavelength was measured using a vacuum FTIR (Bruker IFS 66 v/S) with a HgCdTe photodetector and a resolution of 0.125 cm^−1^. Far field distribution patterns were obtained with a computer-controlled rotational stage and a mercury cadmium telluride (MCT) detector.

## Data Availability

The datasets generated during and/or analysed during the current study are available from the corresponding author on reasonable request.
